# Tempol Attenuates Methotrexate-Induced Osteotoxicity via Antioxidant Mechanisms: Impairment of Protection by GPX4 Inhibition Through ML210

**DOI:** 10.3390/cimb48030326

**Published:** 2026-03-19

**Authors:** Osman Fatih Arpağ, Fariz Selimli, Ahmet Can Haskan, Muhammed Said Altun, Soner Mete, Halil Mahir Kaplan

**Affiliations:** 1 Department of Periodontology, Faculty of Dentistry, Hatay Mustafa Kemal University, 31001 Hatay, Turkey; 2 Department of Oral and Maxillofacial Surgery, Faculty of Dentistry, Hatay Mustafa Kemal University, 31001 Hatay, Turkey; ahmtcnhskn@gmail.com (A.C.H.); msa.said94@gmail.com (M.S.A.); 3 Medical Promotion and Marketing Program, Department of Medical Services and Techniques, Medical Vocational Higher Services School, Nevsehir Hacibektas University, 50300 Nevşehir, Turkey; sonermete@nevsehir.edu.tr; 4 Department of Medicinal Pharmacology, Faculty of Medicine, Cukurova University, 01330 Adana, Turkey; mkaplan@cu.edu.tr

**Keywords:** Tempol, Methotrexate, osteotoxicity, oxidative stress, GPX4, ML210, MAPK, antioxidant therapy

## Abstract

Purpose: Osteotoxicity is a well-recognized adverse effect of Methotrexate (MTX) therapy, primarily driven by oxidative stress and impaired bone remodeling. This study aimed to investigate the protective effects of Tempol, a membrane-permeable nitroxide antioxidant, against MTX-induced osteotoxicity, and to assess how these effects are influenced by ML210, a glutathione peroxidase 4 (GPX4) inhibitor. Methods: Murine osteocyte-like MLO-Y4 cells were treated with MTX alone, Tempol alone, or a combination of MTX with Tempol and ML210. Apoptotic markers (caspase-3, Bax, Bcl-2), MAPK signaling proteins (p-JNK, p-ERK), and oxidative stress parameters (TAS, TOS, SOD, GPx) were measured via ELISA to evaluate the redox and apoptotic responses. Results: MTX significantly induced apoptosis, as evidenced by increased caspase-3 activity and Bax expression, along with decreased Bcl-2 levels. MTX also activated the MAPK pathway by upregulating p-JNK and p-ERK. Furthermore, MTX decreased TAS, SOD, and GPx levels, while increasing TOS. Tempol treatment successfully reversed these effects, restoring apoptotic balance, inhibiting MAPK activation, and enhancing antioxidant capacity. However, co-treatment with ML210 markedly attenuated Tempol’s protective effects, resulting in sustained oxidative stress, elevated apoptotic markers, and persistent MAPK pathway activation. This suggests that Tempol’s cytoprotective actions are dependent on functional GPX4 activity. Conclusion: Tempol exhibits strong potential as an adjunctive antioxidant therapy to counteract MTX-induced osteotoxicity. Nevertheless, its efficacy is significantly influenced by the status of the endogenous antioxidant enzyme GPX4. These findings underscore the need for further investigation into Tempol’s mechanism of action in redox-dependent pathways and its suitability in clinical settings, especially where GPX4 function may be compromised.

## 1. Introduction

Osteotoxicity, also known as skeletal toxicity, refers to the detrimental effects exerted on skeletal tissues during their development and homeostatic maintenance. Such effects may result from exposure to various exogenous substances collectively termed osteotoxicants which can be encountered through environmental contact, ingestion, or injury. These agents may disrupt fundamental skeletal processes such as bone mineralization, remodeling, and morphogenesis, ultimately leading to skeletal deformities and structural weaknesses [1,2].

Methotrexate (MTX), a commonly used antifolate chemotherapeutic and immunosuppressive agent, is well-documented for its osteotoxic potential. MTX impairs osteoblast proliferation and differentiation, disrupts normal bone formation, and simultaneously enhances osteoclastogenesis, resulting in an imbalance that leads to bone demineralization and increased fracture risk [2,3]. At the molecular level, MTX-induced oxidative stress plays a critical role in this process, where elevated reactive oxygen species (ROS) drive pro-apoptotic and inflammatory signaling cascades, particularly involving the Mitogen-Activated Protein Kinase (MAPK) pathway [3,4]. This pathway is activated in response to oxidative stress and contributes to altered bone cell fate and function [3,5].

Tempol (TPL), a stable nitroxide radical and superoxide dismutase (SOD) mimetic, has shown promise in alleviating oxidative damage through free radical scavenging and redox modulation [3,6,7]. At therapeutic doses, TPL mitigates ROS accumulation and prevents hydroxyl radical formation, protecting tissues from oxidative injury [8]. In preclinical models, TPL has demonstrated protective effects against MTX-induced cellular stress and bone loss, partly by suppressing MAPK activation [3]. However, TPL’s dual action as both antioxidant and, at high concentrations, pro-oxidant, necessitates careful dose-dependent evaluation in therapeutic contexts.

Recent studies have explored additional molecular players involved in oxidative stress-mediated bone damage, one of which is Glutathione Peroxidase 4 (GPX4) a crucial enzyme that protects cells from lipid peroxidation and ferroptosis. ML210, a potent and selective covalent inhibitor of GPX4, induces ferroptotic cell death by suppressing lipid peroxide detoxification [9,10]. While ferroptosis has been largely studied in cancer biology, its potential involvement in bone pathology, particularly under oxidative stress conditions such as those induced by MTX, is gaining interest. The inhibition of GPX4 by ML210 increases intracellular ROS and lipid peroxidation, offering a unique model to study how excessive oxidative damage contributes to osteotoxicity [10].

Combining MTX with ML210 in experimental models allows for the dissection of both apoptotic and ferroptotic mechanisms of bone cell death. Furthermore, the co-administration of Tempol may provide insights into how antioxidant defense systems can counteract not only MTX-induced oxidative damage but also lipid peroxidation-driven ferroptosis promoted by GPX4 inhibition. This tri-compound interaction provides a comprehensive platform to investigate redox biology in osteotoxicity and the therapeutic potential of oxidative stress modulators.

This study aims to investigate the protective mechanisms of Tempol against Methotrexate-induced osteotoxicity and to determine how this protection is influenced by GPX4 inhibition via ML210. The findings suggest that although Tempol effectively reduces oxidative stress and modulates MAPK signaling to protect bone cell integrity, its protective capacity is markedly reduced when co-administered with ML210, indicating that the enzymatic activity of GPX4 plays a pivotal role in maintaining the redox homeostasis required for Tempol’s efficacy. ML210-induced suppression of GPX4 appears to increase lipid peroxidation and disrupt cellular antioxidant defenses, thereby diminishing Tempol’s ability to counteract Methotrexate toxicity. This study differs from prior work by (i) evaluating Tempol in MLO-Y4 osteocyte-like cells rather than osteoblasts, (ii) testing the dependence of Tempol’s protective effects on GPX4 using the selective inhibitor ML210, and (iii) integrating MAPK/apoptotic signaling with GPX4-dependent redox regulation to propose a mechanistic link between oxidative stress and osteocyte injury. These results offer valuable insight into the complex interplay between oxidative stress, lipid metabolism, and bone cell vulnerability during MTX treatment.

## 2. Materials and Methods

### 2.1. Study Location and Duration

This experimental study was conducted in the Department of Pharmacology at the Faculty of Medicine, Çukurova University, between 1 January 2023, and 1 May 2024.

### 2.2. Reagents and Materials

All cell culture media used in this research were procured from GIBCO BRL (Grand Island, NY, USA). Calf serum was obtained from HyClone Laboratories (Logan, UT, USA), and rat tail collagen type I was supplied by Becton Dickinson (Bedford, MA, USA). Additional reagents such as fetal bovine serum, bovine serum albumin, phosphate-buffered saline (PBS), sodium chloride (NaCl), Triton X-100, EGTA (ethylene glycol tetraacetic acid), dithiothreitol, sodium fluoride (NaF), Tris-HCl, ML210 and sodium orthovanadate (Na_3_VO_4_) were purchased from Sigma-Aldrich (St. Louis, MO, USA). ELISA kits specific for bax, bcl-2, wee1, AIF, gadd153, and grp78 proteins were obtained from Shanghai Sunred Biological Technology Co., Ltd. (Shanghai, China). Assays for phosphorylated ERK (p-ERK) and JNK (p-JNK) were provided by MyBioSource (San Diego, CA, USA). For antioxidant measurements, SOD and GPx activity kits were purchased from BioVision (Milpitas, CA, USA), and TAS/TOS kits were sourced from Rel Assay Diagnostics (Gaziantep, Turkey). Total protein levels were determined using the Bradford reagent from Bio-Rad Laboratories (Hercules, CA, USA).

MTX (10^−5^ M), Tempol (100 µM) and ML210 (1 µM) were chosen based on prior studies in similar cell types and our preliminary dose–response experiments [3].

### 2.3. Cell Line and Culture Conditions

The murine osteocyte-like cell line MLO-Y4 was acquired from Kerafast, Inc. (Boston, MA, USA) and cultured according to the methods described by Kato and Bonewald [11]. Cells were treated with Methotrexate (MTX) at a concentration of 10^−5^ M for 48 h, either alone or in combination with Tempol at 100 μM. For triple treatment conditions, ML210 was added at a final concentration of 1 μM alongside MTX and Tempol. Following treatment, cells were processed for downstream analysis.

### 2.4. Cell Homogenization Procedure

Following 48-h treatments in six-well plates, cells were collected into 15 mL tubes. Samples were centrifuged at 2000 rpm for 10 min at 4 °C, and the supernatant was removed. Cells were washed with 5 mL PBS and centrifuged again under the same conditions. The pellets were then resuspended in 250 μL RIPA buffer, supplemented with 2.5 μL of PMSF (200 mM), 2.5 μL sodium orthovanadate (100 mM), and 2.5 μL protease inhibitor cocktail. Cell lysis was performed using an ultrasonic homogenizer on ice. After homogenization, samples were centrifuged at 10,000 rpm for 10 min. The resulting supernatants were stored for analysis, while the pellets were discarded.

### 2.5. Total Protein Quantification

Total protein concentrations were determined by the Bradford assay using bovine serum albumin (BSA) standards (1–100 μg/mL) to generate a standard curve. Samples and standards were assayed in duplicate, absorbance was read at 595 nm, and sample concentrations were calculated from the standard curve using GraphPad Prism. (ver. 9.0) Measured protein concentrations were reported per μg total protein for normalization of biochemical assays. For quality control, blank subtraction was performed, standard-curve linearity was checked, and samples with a coefficient of variation > 15% were remeasured. Protein concentrations were calculated using Prism software.

### 2.6. ELISA (Enzyme-Linked Immunosorbent Assay) Tests

The ELISA technique was employed to measure caspase-3 activity and the expression levels of proteins involved in apoptotic pathways, including bax and bcl-2. Additionally, active ERK (p-ERK) and JNK (p-JNK) were analyzed via ELISA. Assessments of TAS, TOS, SOD, and GPx were carried out following previously described methodologies.

### 2.7. Statistical Analysis

Data are expressed as means ± S.E.M., with “n” denoting the number of cell culture flasks utilized for each group. Comparisons between the groups were made using analysis of variance (ANOVA) with Bonferroni corrections applied for multiple comparisons, and *p*-values less than 0.05 were deemed statistically significant.

## 3. Results

In this study, we examined key mediators of apoptosis to elucidate the mechanisms underlying Methotrexate (MTX)-induced osteotoxicity and the potential modulatory effects of Tempol and ML210. Apoptosis was evaluated via caspase-3 activity and the expression levels of regulatory proteins such as bax, bcl-2, wee1, grp78, gadd153, and AIF. Caspase-3 and bax are known to promote apoptotic cell death, while bcl-2 acts as an anti-apoptotic factor [12].

**Figure 1:**
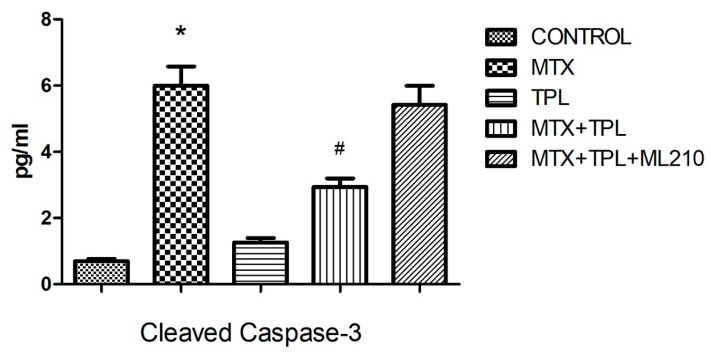
Effect of MTX, TPL, and ML210 on caspase-3 activity (n = 8). Caspase-3 activity significantly increased in the MTX group compared to control, while TPL reduced this effect. However, co-treatment with ML210 attenuated the protective effect of TPL. Statistical analysis: ANOVA. Post hoc: Bonferroni. (n:6, *: vs. control, *p* < 0.05; #: vs. MTX, *p* < 0.05).

**Figure 2:**
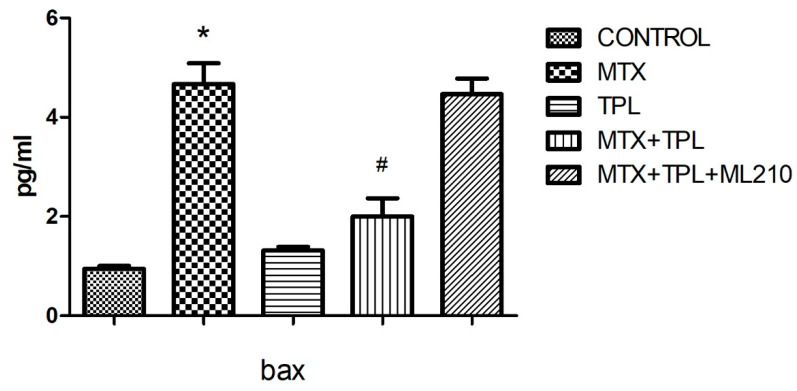
Effect of MTX, TPL, and ML210 on bax protein levels (n = 8). Bax expression increased after MTX treatment; TPL suppressed this upregulation, while ML210 reversed the suppression induced by TPL. Statistical analysis: ANOVA. Post hoc: Bonferroni. (n:6, *: vs. control, *p* < 0.05; #: vs. MTX, *p* < 0.05).

**Figure 3:**
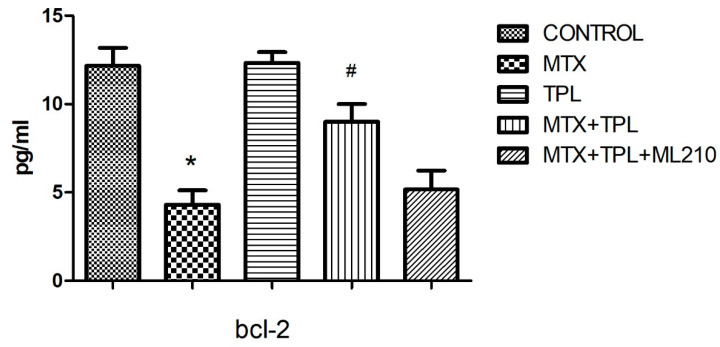
Effect of MTX, TPL, and ML210 on bcl-2 levels (n = 8). MTX reduced bcl-2 expression, which was restored by TPL. However, the addition of ML210 significantly weakened this restorative effect. Statistical analysis: ANOVA. Post hoc: Bonferroni. (n:6, *: vs. control, *p* < 0.05; #: vs. MTX, *p* < 0.05).

Our data revealed that MTX treatment significantly increased caspase-3 activity (Figure 1, *p* < 0.0001) and bax expression (Figure 2, *p* < 0.0001), accompanied by a marked reduction in bcl-2 levels (Figure 3, *p* < 0.0001). These changes disrupted the bax/bcl-2 ratio, thereby favoring apoptosis. Additionally, the expression of protective or regulatory proteins, including wee1, grp78, gadd153, and AIF, was significantly reduced in MTX-treated cells (Table 1), further indicating cellular susceptibility to death signaling.

Tempol treatment, when administered alongside MTX, significantly mitigated these alterations, restoring bax/bcl-2 balance and enhancing the expression of protective proteins. However, co-treatment with ML210 markedly blunted Tempol’s protective effects. Specifically, in the MTX + Tempol + ML210 group, caspase-3 and bax levels remained significantly elevated, and bcl-2 expression remained suppressed compared to the MTX + Tempol group (*p* < 0.0001, all comparisons). This suggests that ML210, via GPX4 inhibition, compromises the anti-apoptotic and redox-regulating capacity of Tempol.

**Figure 4:**
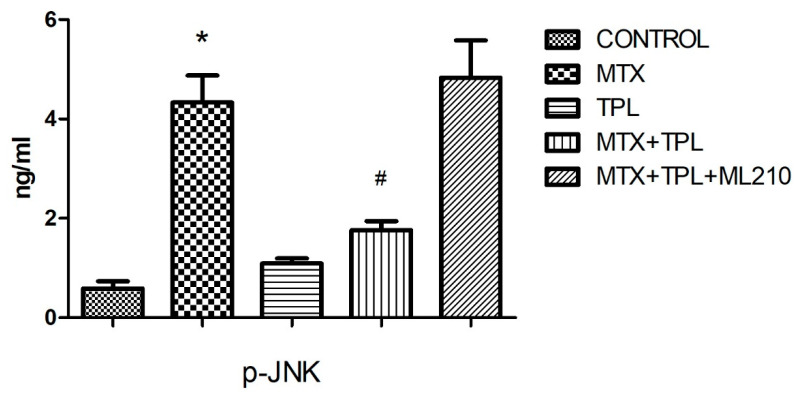
Effect of MTX, TPL, and ML210 on JNK (p-JNK) activity (n = 8). MTX induced JNK activation, while TPL significantly reduced p-JNK levels. ML210 co-treatment limited this suppression. Statistical analysis: ANOVA. Post hoc: Bonferroni. (n:6, *: vs. control, *p* < 0.05; #: vs. MTX, *p* < 0.05).

**Figure 5:**
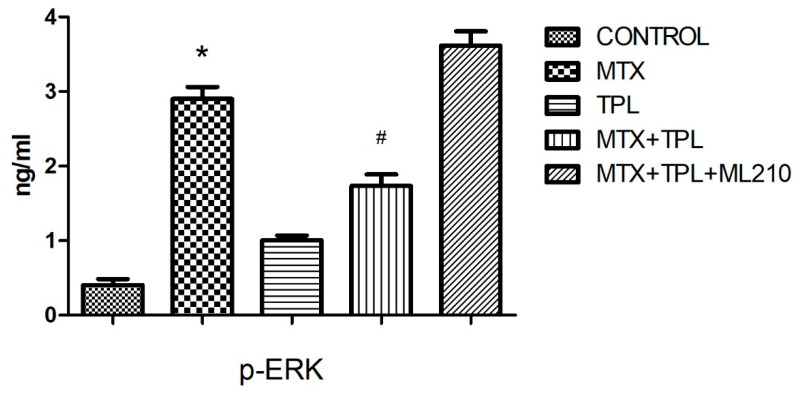
Effect of MTX, TPL, and ML210 on ERK (p-ERK) activity (n = 8). ERK phosphorylation was elevated by MTX and mitigated by TPL; this effect was partially reversed when ML210 was co-administered. Statistical analysis: ANOVA. Post hoc: Bonferroni. (n:6, *: vs. control, *p* < 0.05; #: vs. MTX, *p* < 0.05).

We also investigated the MAPK signaling pathway, focusing on phosphorylated JNK and ERK, which are central regulators of stress-induced signaling [3,13]. MTX exposure led to pronounced activation of both p-JNK and p-ERK, indicating heightened stress response (Figure 4 and Figure 5, *p* < 0.0001). Tempol significantly suppressed this activation. However, in the presence of ML210, the inhibitory effect of Tempol on MAPK activation was partially reversed, with both p-JNK and p-ERK levels remaining significantly higher than in the Tempol-only group, but lower than MTX-alone (*p* < 0.0001, all groups), indicating incomplete rescue.

**Figure 6:**
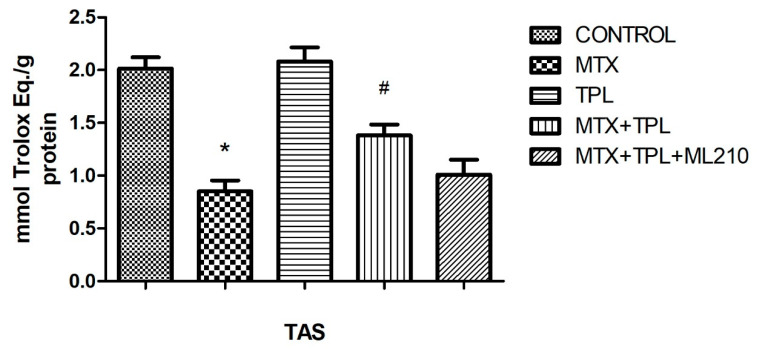
Effect of MTX, TPL, and ML210 on Total Antioxidant Status (TAS) (n = 8). MTX reduced TAS levels, which were restored by TPL; ML210 co-treatment prevented full restoration. Statistical analysis: ANOVA. Post hoc: Bonferroni. (n:6, *: vs. control, *p* < 0.05; #: vs. MTX, *p* < 0.05).

**Figure 7:**
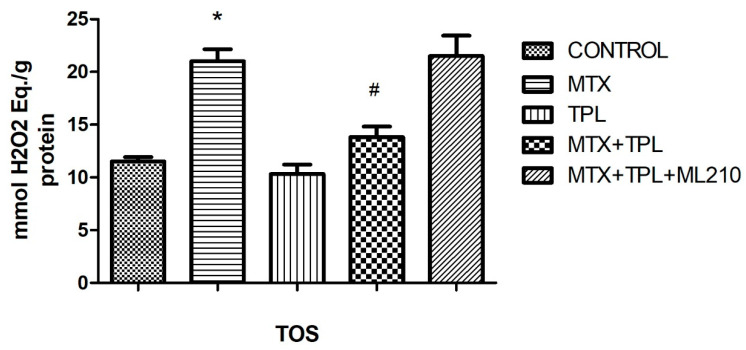
Effect of MTX, TPL, and ML210 on Total Oxidant Status (TOS) (n = 8). TOS levels increased in MTX-treated cells and were reduced by TPL. ML210 treatment diminished this antioxidant effect. Statistical analysis: ANOVA. Post hoc: Bonferroni. (*: vs. control, *p* < 0.05; #: vs. MTX, *p* < 0.05).

In assessing oxidative stress markers, MTX significantly reduced Total Antioxidant Status (TAS) levels (Figure 6, *p* < 0.0001), while Total Oxidant Status (TOS) levels were elevated (Figure 7, *p* < 0.0001). Tempol treatment effectively reversed these changes, increasing TAS and lowering TOS. However, ML210 co-treatment diminished these antioxidant effects, with TAS remaining suppressed and TOS elevated compared to the MTX + Tempol group (*p* < 0.0001).

**Figure 8:**
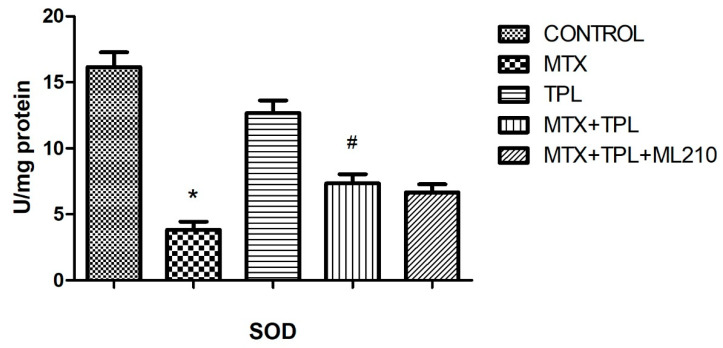
Effect of MTX, TPL, and ML210 on superoxide dismutase (SOD) activity (n = 8). MTX suppressed SOD activity, which was restored by TPL; however, ML210 interfered with this restoration. Statistical analysis: ANOVA. Post hoc: Bonferroni. (n:6, *: vs. control, *p* < 0.05; #: vs. MTX, *p* < 0.05).

**Figure 9:**
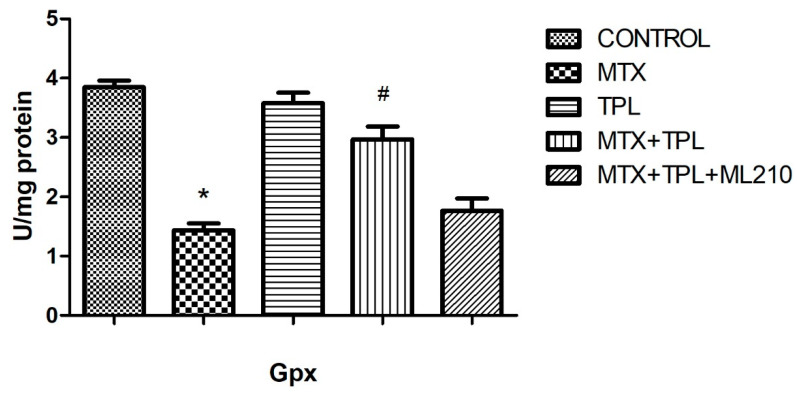
Effect of MTX, TPL, and ML210 on glutathione peroxidase (GPx) activity (n = 8). GPx activity declined following MTX exposure and improved with TPL treatment. This effect was notably impaired by ML210. Statistical analysis: ANOVA. Post hoc: Bonferroni. (n:6, *: vs. control, *p* < 0.05; #: vs. MTX, *p* < 0.05).

Moreover, the activities of two critical antioxidant enzymes superoxide dismutase (SOD) and glutathione peroxidase (GPx) were significantly decreased in the MTX group (Figure 8 and Figure 9, *p* < 0.0001). Tempol restored both enzymes to near-control levels. Yet, in the MTX + Tempol + ML210 group, SOD and GPx activity remained significantly lower, indicating that ML210-mediated GPX4 inhibition impairs Tempol’s ability to restore antioxidant defense mechanisms.

Collectively, these findings indicate that while Tempol exerts strong protective effects against MTX-induced oxidative damage and apoptosis, these effects are significantly compromised by the presence of ML210, highlighting the crucial role of GPX4 in maintaining redox homeostasis and supporting Tempol’s efficacy.

## 4. Discussion

### 4.1. Apoptotic Mediators

This study investigated the pro-apoptotic and protective mechanisms in Methotrexate (MTX)-induced osteotoxicity, particularly focusing on the modulation of key apoptotic markers. As expected, MTX exposure led to a marked increase in caspase-3 activity and bax expression, along with a significant decrease in bcl-2, disrupting the bax/bcl-2 balance and favoring cell death pathways [3,14,15]. This confirms MTX’s well-established role in promoting programmed cell death via intrinsic apoptotic mechanisms.

Tempol (TPL), a stable nitroxide compound with SOD mimetic properties, demonstrated a notable protective effect in the MTX-treated cells. TPL treatment restored bcl-2 expression, suppressed bax and caspase-3, and preserved the viability of osteocyte-like cells. These protective effects are likely attributed to TPL’s ability to scavenge superoxide radicals (O_2_•^−^), reduce ferrous iron (Fe^2+^) availability, and subsequently limit hydroxyl radical (•OH) generation [3]. However, this beneficial profile was substantially compromised when ML210 was introduced in combination with TPL.

ML210, a covalent GPX4 inhibitor, is known to suppress lipid peroxide detoxification, leading to accumulation of lipid ROS and iron-dependent cell death mechanisms resembling ferroptosis [16]. In our findings, the MTX + TPL + ML210 group exhibited elevated caspase-3 and bax levels, and suppressed bcl-2 expression, indicating that the protective anti-apoptotic effects of TPL were effectively abrogated by GPX4 inhibition. This suggests that GPX4 activity is essential for Tempol to exert its full antioxidant and cytoprotective function.

Moreover, other apoptotic markers such as wee1, gadd153, grp78, and AIF were also significantly downregulated in MTX-treated cells, and partially rescued by TPL. However, in the presence of ML210, these restorative effects were lost, reinforcing the concept that GPX4 is a central regulator of oxidative and apoptotic homeostasis.

### 4.2. MAPK Signaling Pathways

The role of ROS in modulating intracellular signaling, particularly via the MAPK pathway, has been well documented. MTX treatment activated both JNK (p-JNK) and ERK (p-ERK) pathways, indicating a stress response that can lead to cell death or survival depending on context [3,17]. While JNK is typically associated with pro-apoptotic responses, ERK can exert dual roles, including survival signaling [18,19].

Tempol’s ability to inhibit MTX-induced MAPK activation was evident in our study, aligning with its antioxidant activity. However, ML210 reversed this inhibitory effect, with p-JNK and p-ERK levels significantly elevated in the MTX + TPL + ML210 group. This suggests that oxidative stress induced by GPX4 inhibition surpasses Tempol’s ROS-scavenging capacity, leading to sustained activation of MAPK pathways and promotion of apoptotic signaling.

### 4.3. Oxidative Stress Parameters

The oxidative stress profile in MTX-treated cells was characterized by decreased Total Antioxidant Status (TAS), increased Total Oxidant Status (TOS), and suppressed SOD and GPx activity. These findings confirm that MTX induces severe oxidative imbalance, contributing to cellular injury and dysfunction.

Tempol effectively reversed these alterations by increasing TAS and enzymatic antioxidant activities (SOD, GPx), while reducing TOS. This underscores its utility in mitigating MTX-induced oxidative damage. However, ML210 co-treatment diminished these protective effects, with TAS levels remaining low, TOS elevated, and SOD/GPx activities significantly suppressed. These results highlight that GPX4 is a key downstream effector of the antioxidant response and that its inhibition by ML210 renders Tempol’s antioxidant defense insufficient to prevent oxidative injury.

### 4.4. Overall Interpretation

This study highlights that the protective effects of Tempol against Methotrexate (MTX)-induced oxidative stress and apoptosis are primarily driven by its potent antioxidant properties. By scavenging superoxide radicals and restoring intracellular redox balance, Tempol effectively reduces reactive oxygen species (ROS) levels and prevents oxidative damage, thereby preserving cell viability.

However, this antioxidant protection is shown to be critically dependent on the activity of glutathione peroxidase 4 (GPX4). Inhibition of GPX4 by ML210 compromises Tempol’s capacity to neutralize oxidative stress, resulting in elevated ROS accumulation, disrupted redox signaling, and increased apoptotic cell death. These findings suggest that GPX4 functions as a key facilitator of Tempol’s redox-regulating actions.

Although GPX4 inhibition by ML210 attenuated Tempol’s protective effects and increased oxidative damage, direct ferroptosis-specific evidence (lipid peroxidation profiles, ACSL4/SLC7A11/FSP1 expression, and rescue by ferroptosis inhibitors) was not comprehensively obtained in the current experiments. Therefore, our data indicate that GPX4-dependent redox regulation contributes to MTX-induced oxidative injury and to Tempol’s protective mechanism, but do not definitively demonstrate ferroptosis. Future work will include lipid peroxidation assays (BODIPY-C11/MDA), ferroptosis markers, and ferroptosis inhibitor rescue experiments to confirm ferroptotic involvement.

Apoptotic markers (caspase-3, BAX, BCL-2) were altered, indicating apoptotic contribution; because ferroptosis is typically caspase-independent, the relative contributions of apoptosis and ferroptosis remain unresolved in this study.

Therefore, when considering antioxidant-based therapies in conjunction with pro-oxidant or cytotoxic agents like MTX, it is essential to evaluate the functional state of cellular antioxidant systems, particularly GPX4. The efficacy of Tempol appears to rely not only on its intrinsic chemical properties but also on the integrity of endogenous antioxidant pathways. Further investigations into the molecular interplay between Tempol, GPX4, and ROS are necessary to refine therapeutic strategies and ensure effective redox-based interventions in oxidative stress-related conditions. The findings provide a molecular basis for evaluating Tempol as a co-therapy to minimize chemotherapy-induced bone side effects.

## 5. Conclusions

In conclusion, this study demonstrates that Tempol exerts significant protective effects against Methotrexate (MTX)-induced oxidative stress and apoptosis in osteocyte-like cells, primarily through its antioxidant capacity and redox-regulating properties. Tempol was shown to restore antioxidant enzyme activity, suppress pro-apoptotic signaling, and modulate MAPK pathway activation, thereby preserving cellular integrity under MTX-induced stress conditions.

However, the protective efficacy of Tempol is markedly compromised in the presence of GPX4 inhibition by ML210. The loss of GPX4 activity led to an accumulation of lipid peroxides and oxidative damage that overwhelmed Tempol’s antioxidant defenses, indicating that GPX4 is essential for maintaining the redox environment required for Tempol’s function.

These findings highlight the importance of considering the cellular redox status and antioxidant system integrity, particularly GPX4, when evaluating or designing antioxidant-based therapies. Tempol holds promise as a supportive agent in mitigating chemotherapy-induced bone toxicity, but its effectiveness may be contingent upon the preservation of endogenous antioxidant mechanisms. Future studies should explore the therapeutic potential of Tempol in redox-sensitive disease models and further define its interaction with ferroptosis-related pathways.

## Figures and Tables

**Table 1:** Effect of MTX, TPL and ML210 treatment on the levels of apoptotic mediators (n = 6, *: *p* < 0.0001 according to control, #: *p* < 0.0001 according to MTX).

	Control	MTX	TPL	MTX + TPL	MTX + TPL + ML210
wee 1 (*p* < 0.0001, n:6)	0.41 ± 0.04 pg/mL	1.26 ± 0.05 pg/mL *	0.35 ± 0.06 pg/mL	0.79 ± 0.05 pg/mL #	1.38 ± 0.06 pg/mL
AIF (*p* < 0.0001, n:6)	0.90 ± 0.05 pg/mL	2.0 ± 0.05 pg/mL *	0.98 ± 0.04 pg/mL	1.4 ± 0.05 pg/mL #	2.2 ± 0.07 pg/mL
gadd153 (*p* < 0.0001, n:6)	0.34 ± 0.03 pg/mL	0.95 ± 0.08 pg/mL *	0.23 ± 0.04 pg/mL	0.70 ± 0.02 pg/mL #	1.05 ± 0.06 pg/mL
grp78 (*p* < 0.0001, n:6)	0.36 ± 0.03 pg/mL	2.2 ± 0.05 pg/mL *	0.45 ± 0.08 pg/mL	1.31 ± 0.05 pg/mL #	1.8 ± 0.03 pg/mL

## Data Availability

The original contributions presented in this study are included in the article. Further inquiries can be directed to the corresponding authors.

## References

[1] Chen Y.Q., Yu T., Song Z.Q., Wang C.Y., Luo J.T., Xiao Y., Qiu H., Wang Q.Q., Jin H.M. (2025). Application of Large Language Models in Drug-Induced Osteotoxicity Prediction. J. Chem. Inf. Model..

[2] Uruç V., Salimov F., Kaplan H.M. (2020). Protective Effect of Extract on Methotrexate-Induced Osteotoxicity via Reducing Oxidative Stress and MAPK Activity. Int. J. Pharmacol..

[3] Selimli F., Reyhanioglu M.T., Haskan A.C., Altun M.S., Mete S., Kaplan H.M. (2025). Tempol Mitigates Methotrexate-Induced Osteotoxicity via Oxidative Stress Modulation and MAPK Pathway Inhibition. Drug Des. Dev. Ther..

[4] Tamhane M., Chakilam A.R., Jayaraj A., Thakkar V., Taft D.R. (2010). Comparative renal excretion of VX-702, a novel p38 MAPK inhibitor, and methotrexate in the perfused rat kidney model. Drug Dev. Ind. Pharm..

[5] Takashima Y., Hayano A., Yamanaka R. (2020). Metabolome Analysis Reveals Excessive Glycolysis via PI3K/AKT/mTOR and RAS/MAPK Signaling in Methotrexate-Resistant Primary CNS Lymphoma-Derived Cells. Clin. Cancer Res..

[6] Santos F.R., Rossetto I.M.U., Montico F., Lamas C.D., Cagnon V.H.A. (2024). Differential tempol effects in prostatic cancer: Angiogenesis and short- and long-term treatments. J. Mol. Histol..

[7] Pinar N., Kaplan M., Ozgur T., Ozcan O. (2018). Ameliorating effects of tempol on methotrexate-induced liver injury in rats. Biomed. Pharmacother..

[8] Du J., Venkataraman S., Oberle L.W., Cullen J.J. (2006). Tempol as a superoxide scavenger in pancreatic cancer. Free Radic. Biol. Med..

[9] Li Y.C., Xiong Y.M., Long Z.P., Huang Y.P., Shu Y.B., He K., Sun H.Y., Shi Z. (2025). ML210 Antagonizes ABCB1- Not ABCG2-Mediated Multidrug Resistance in Colorectal Cancer. Biomedicines.

[10] Wang H., Wang C., Li B.R., Zheng C.X., Liu G.Q., Liu Z.M., Zhang L.R., Xu P. (2023). Discovery of ML210-Based glutathione peroxidase 4 (GPX4) degrader inducing ferroptosis of human cancer cells. Eur. J. Med. Chem..

[11] Bonewald L.F. (1999). Establishment and characterization of an osteocyte-like cell line, MLO-Y4. J. Bone Min. Metab..

[12] Celik E., Kaplan H.M., Singirik E. (2020). The impact of propranolol on apoptosis in cutaneous squamous cell carcinomas. Bratisl. Med. J.-Bratisl. Lek. Listy.

[13] Okyay A.G., Kaplan H.M., Asil H., Singirik E. (2020). Saffron induces Apoptosis in Ovarian Cancer cell via MAPK and AKT/mTOR Pathways. Prog. Nutr..

[14] El-Dessouki A.M., Kaml M.E., EL-Yamany M.F. (2025). Modulation of AMPK by esomeprazole and canagliflozin mitigates methotrexate-induced hepatotoxicity: Involvement of MAPK/JNK/ERK, JAK1/STAT3, and PI3K/Akt signaling pathways. Naunyn-Schmiedeberg’s Arch. Pharmacol..

[15] Kaymak E., Ceylan T., Akin T., Kuloglu N., Sayan M., Degen N., Önal E., Yildirim A.B., Karabulut D. (2024). Effect of thymoquinone on NRF2/NF-kB/MAPK pathway in methotrexate-induced rat testis injury. Iran. J. Basic Med. Sci..

[16] Kudryashova O.M., Nesterenko A.M., Korzhenevskii D.A., Sulyagin V.K., Tereshchuk V.M., Belousov V.V., Shokhina A.G. (2023). Proteomic Shift in Mouse Embryonic Fibroblasts Pfa1 during Erastin, ML210, and BSO-Induced Ferroptosis. Data.

[17] Wu T., Yu Y., Tu X., Ye L., Wang J., Xie C., Kuang K., Yu Y., Zhuge W., Wang Z. (2025). Tubeimoside-I, an inhibitor of HSPD1, enhances cytotoxicity of oxaliplatin by activating ER stress and MAPK signaling pathways in colorectal cancer. J. Ethnopharmacol..

[18] du Plessis J., Deroubaix A., Omar A., Penny C. (2024). A Bioinformatic Analysis Predicts That Cannabidiol Could Function as a Potential Inhibitor of the MAPK Pathway in Colorectal Cancer. Curr. Issues Mol. Biol..

[19] Di Y., Li L., Xu J., Liu A., Zhao R., Li S., Li Y., Ding J., Chen S., Qu M. (2024). MAPK signaling pathway enhances tolerance of Mytilus galloprovincialis to co-exposure of sulfamethoxazole and polyethylene microplastics. Environ. Pollut..

